# Harvesting the Power of Green Synthesis: Gold Nanoparticles Tailored for Prostate Cancer Therapy

**DOI:** 10.3390/ijms25042277

**Published:** 2024-02-14

**Authors:** Marco Oliveira, André Sousa, Sara Sá, Sílvia Soares, Ana Cláudia Pereira, Ana Catarina Rocha, Patrick Pais, Diogo Ferreira, Cátia Almeida, Carla Luís, Cláudio Lima, Fábio Almeida, Álvaro Gestoso, Miguel-Correa Duarte, Pedro Barata, Daniela Martins-Mendes, Pilar Baylina, Carla F. Pereira, Rúben Fernandes

**Affiliations:** 1 FP-I3ID, FP-BHS, Instituto de Investigação, Inovação e Desenvolvimento, Biomedical Health Sciences, Universidade Fernando Pessoa (UFP), 4249-004 Porto, Portugal; 2 CECLIN, Centro de Estudos Clínicos, Hospital Escola Fernando Pessoa, 4420-096 Gondomar, Portugal; 3 RISE-UFP, Rede de Investigação em Saúde, Universidade Fernando Pessoa, 4249-004 Porto, Portugal; 4 i3S, Instituto de Investigação e Inovação em Saúde, 4200-135 Porto, Portugal; 5 FMUP, Faculty of Medicine of the University of Porto, 4200-319 Porto, Portugal; 6 FFCC-Facultad de Ciencias, University of Vigo, 36310 Vigo, Spain; 7 ECVA-UTAD, Escola de Ciências da Vida e do Ambiente, Universidade de Trás-os-Montes e Alto Douro, 5000-801 Vila Real, Portugal; 8 TBIO, Center for Translational Health and Medical Biotechnology Research, ESS-IPP, Escola S. Saúde, Instituto Politécnico do Porto, 4200-465 Porto, Portugal; 9 CINBIO, University of Vigo, 36310 Vigo, Spain; 10 Southern Galicia Institute of Health Research (IISGS), Biomedical Research Networking Center for Mental Health (CIBERSAM), 36310 Madrid, Spain

**Keywords:** biosynthetic gold nanoparticles, *P. aeruginosa*, prostate cancer, anticancer activity

## Abstract

Biosynthetic gold nanoparticles (bAuNPs) present a promising avenue for enhancing bio-compatibility and offering an economically and environmentally responsible alternative to traditional production methods, achieved through a reduction in the use of hazardous chemicals. While the potential of bAuNPs as anticancer agents has been explored, there is a limited body of research focusing on the crucial physicochemical conditions influencing bAuNP production. In this study, we aim to identify the optimal growth phase of *Pseudomonas aeruginosa* cultures that maximizes the redox potential and coordinates the formation of bAuNPs with increased efficiency. The investigation employs 2,6-dichlorophenolindophenol (DCIP) as a redox indicator. Simultaneously, we explore the impact of temperature, pH, and incubation duration on the biosynthesis of bAuNPs, with a specific emphasis on their potential application as antitumor agents. Characterization of the resulting bAuNPs is conducted using ATR-FT-IR, TEM, and UV-Vis spectroscopy. To gain insights into the anticancer potential of bAuNPs, an experimental model is employed, utilizing both non-neoplastic (HPEpiC) and neoplastic (PC3) epithelial cell lines. Notably, *P. aeruginosa* cultures at 9 h/OD600 = 1, combined with biosynthesis at pH 9.0 for 24 h at 58 °C, produce bAuNPs that exhibit smaller, more spherical, and less aggregated characteristics. Crucially, these nanoparticles demonstrate negligible effects on HPEpiC cells while significantly impacting PC3 cells, resulting in reduced viability, migration, and lower IL-6 levels. This research lays the groundwork for the development of more specialized, economical, and ecologically friendly treatment modalities.

## 1. Introduction

Numerous nanomaterials and nanoparticles, synthesized through biological, physical, chemical, or hybrid methods, have emerged using various building materials such as gold, silver, zinc, iron, silica, copper, and cobalt [1,2]. The tuning of nanoparticles’ (NPs) chemical, physical, and biological properties provides versatility for applications in different fields, including serving as image contrast agents, as vectors for drug or small molecule delivery, and for diagnostic purposes [3]. Gold nanoparticles (AuNPs) have gained significance, especially in the biological and pharmaceutical fields, due to their unique optical and physical characteristics suitable for various applications [4].

**Figure 1:**
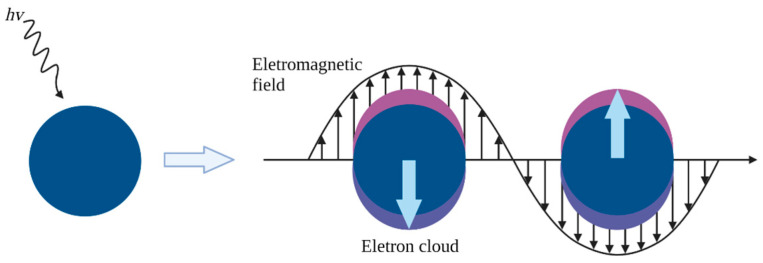
Illustration of the resonance effect of gold electrons in response to incident radiation [8].

The primary optical potential of AuNPs depends on surface plasmon resonance (SPR), a phenomenon involving the dispersion and absorption of light by gold electrons in response to incident radiation (Figure 1). In metallic nanostructures, SPR is described by oscillating electrons with a frequency equal to the irradiating electromagnetic field [5]. The optical properties of AuNPs are highly reliant on their size, shape, and structure. The position and width of the plasmon resonance peak provide information about the average diameter and distribution of particle size [6,7,8].

AuNPs exhibit tunable properties, resulting in various sizes and shapes (nanorods, nanostars, nanocubes, nanocages, and nanospheres, among others), with different yields and dispersity depending on the synthesis parameters [9]. Aggregation between AuNPs can lead to a significant red-shifting of the SPR frequency, causing a change in the solution color from red to blue and widening the surface plasmon band [10]. Similar effects are observed with changes in size [11].

**Figure 2:**
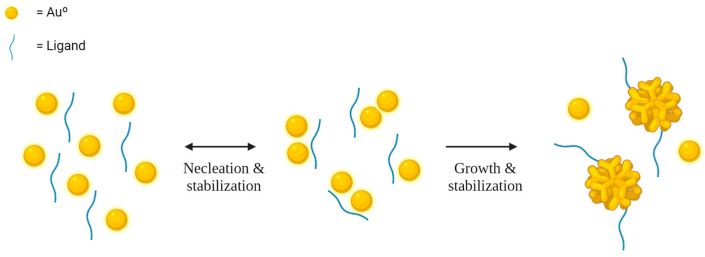
Illustration outlining the general mechanistic steps involved in the synthesis of nanoparticles [14].

Various synthesis methods can be applied to produce AuNPs. Typically, chemical reduction processes, such as the widely used Turkevitch method (Figure 2) [12], involve reducing gold ions to their elemental form using a reducing agent. Physical methods, including radiation (microwave, gamma irradiation, or ultraviolet) and laser ablation, are also employed in AuNPs preparation [13].

The synthesis of AuNPs consists of two main steps: (I) nucleation and (II) growth phase. Nucleation is the formation of clusters in a single-phase system, and in the context of AuNPs synthesis, these clusters are referred to as nuclei. The critical radius of these clusters determines the smallest size at which a particle may remain in solution without redissolving. The growth phase involves depositing more material on the cluster, increasing its size and transforming it into a nanoparticle. This process is governed by the spread of growing species and surface processes [12,14,15]. While chemical synthesis methods for gold nanocrystals have grown in popularity, typically, this approach entails employing environmentally hazardous reagents that are challenging to manage and dispose of due to their toxic, corrosive, irritating, and/or flammable characteristics. For instance, a case in point is the synthesis involving the use of organic or inorganic solvents in combination with reducing agents like sodium borohydride, *N*,*N*-dimethylformamide, and hydrazine, as well as stabilizing agents such as cetrimonium bromide, poly(amidoamine), *N*-methyl-D-glucamine, citric acid, and poly(vinylpyrrolidone), among others [16,17,18,19,20,21].

Within the realm of “green” synthesis methods, bacteria stand out as essential tools for producing NPs due to their diversity and high adaptability to extreme conditions [22]. Bacterial NP synthesis holds great promise, characterized by low energy consumption and process controllability [23]. Metal NPs can be formed by bacteria both intracellularly and extracellularly. Extracellular synthesis has been identified as more efficient and facilitates the extraction of NPs. In this context, biosynthetic metal NPs exhibit enhanced resistance to oxidation, rendering them applicable in various fields [24]. The biosynthesis of nanoparticles involves the oxidation/reduction of metallic ions by biomolecules secreted by microorganisms, leading to the production of biocompatible, bioavailable, bioactive, and bioabsorbable nanoparticles [25].

*Pseudomonas aeruginosa*, despite its pathogenicity, exhibits diverse beneficial roles. It produces compounds like pyocyanin, quinolines, phenylpyrroles, and phenazines with bactericidal properties, showcasing potential in medicine. In various industries, *P. aeruginosa* is explored for applications in oil refineries, waste degradation, textiles, pulp and paper, agriculture, mining, and explosives [26]. Its adaptability makes it valuable for bioremediation and biodegradation in soils contaminated with herbicides and petroleum [27]. *P. aeruginosa* is also investigated for gold nanoparticle (AuNP) production. Studies, such as one by Abd El-Aziz M. et al., highlight its extracellular biosynthesis potential, indicating simplicity and improved downstream processing [28]. Although limited, some studies, like Timoszyk et al.’s work, explore variations in AuNP synthesis conditions using *P. aeruginosa*, revealing size dependence on temperature and antimicrobial activity against *E. coli* [29]. 

**Figure 3:**
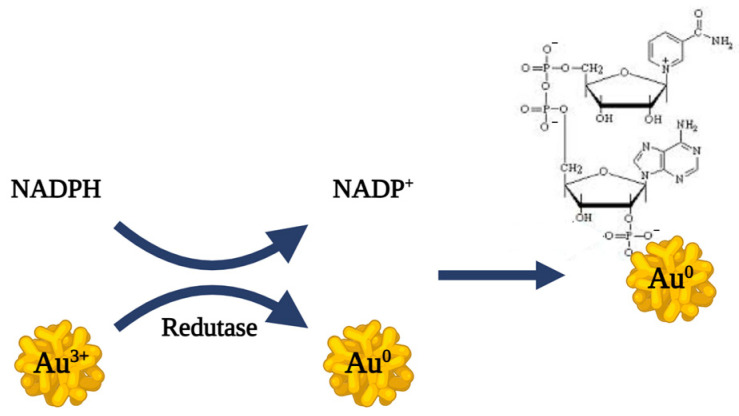
Representation of the putative mechanism of bAuNPs synthesis proposed by Nangia et al. [30].

The biosynthesis mechanism of bAuNPs by *P. aeruginosa* remains unclear. However, Nangia et al. highlighted an NADPH-dependent reductase enzyme that converts Au(III) to Au(0) through an electron shuttle enzymatic metal reduction process in *Stenotrophomonas maltophilia,* a bacterium belonging, like *P. aeruginosa*, to the Gammaproteobacteria class. This suggests the capping of bAuNPs by negatively charged phosphate ions from NADP (Figure 3) [30].

In *P. aeruginosa*, various genes encode NADPH-dependent enzymes related to oxidative stress and antimicrobial susceptibility. One such gene is glutathione reductase (GR). In *E. coli*, glutathione (GSH) peroxidase reduces hydrogen peroxide to water and oxygen molecules, and the two molecules of GSH are oxidized to form GSH disulfide (GSSG). At high concentrations, GSSG is toxic and must be reduced back to GSH using electrons from NADPH through GSH reductase. GSH reductase appears to play a similar role in *P. aeruginosa* [31,32].

Prostate cancer (PCa) is estimated to have the highest worldwide incidence among men, making nanotechnology, particularly in the form of gold nanoparticles (AuNPs), a significant focus in nano-oncology. AuNPs demonstrate anticancer activity through mechanisms such as interacting with cell membranes, inducing apoptosis via reactive oxygen species (ROS), and interfering with the chemistry of proteins or DNA [33]. The inflammatory process, which involves cytokines like interleukin 6 (IL-6), plays a significant role in the pathogenesis of PCa [34,35].

Numerous studies have investigated the cytotoxicity of gold nanoparticles (AuNPs) on normal and cancer cells, highlighting the impact of shape and size. Spherical AuNPs were reported to be more efficiently absorbed and toxic, contrasting with varied results for different shapes [36,37,38]. Surface properties play a crucial role in cellular uptake mechanisms, involving interactions between positive charges on AuNPs and negative charges on cellular membranes [35,39,40].

Cytotoxicity is significantly influenced by AuNP concentration, with varying opinions due to diverse shapes, sizes, and capping agents [41,42,43,44,45]. Prema et al. found antiproliferative effects of green tea-synthesized AuNPs in PC3 cells, attributing it to highly active biomolecules [46]. Nambiar et al. observed an interesting antitumoral effect of curcumin-coated AuNPs in a serum-free environment, but reduced cytotoxicity in the presence of serum protein due to altered surface properties [47].

**Figure 4:**
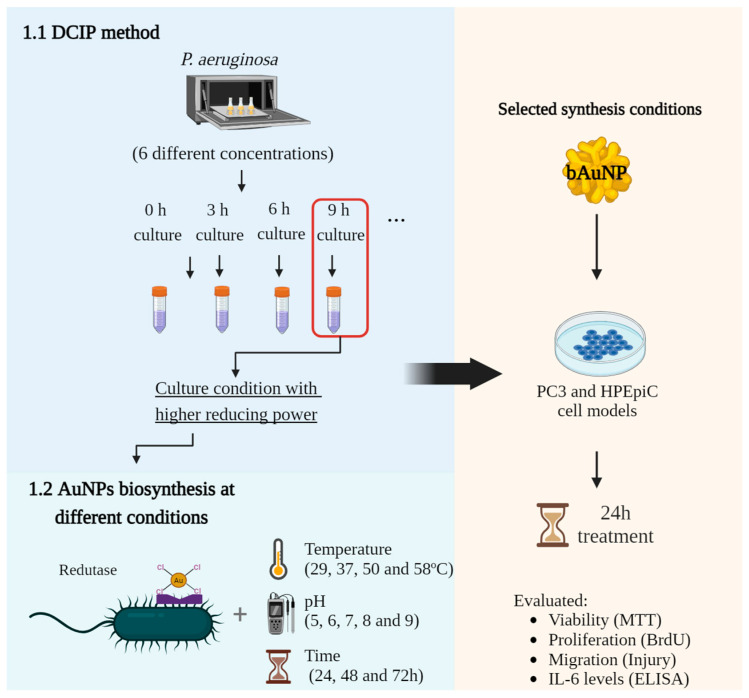
Experimental design overview.

This study aims to improve a simple and cost-effective method for synthesizing green gold nanoparticles using a *P. aeruginosa* strain PAO1 known for its production of bioactive compounds. The primary objectives include determining effective culture conditions, observing the effects of pH, temperature, and incubation time on bAuNPs synthesis, and examining how tuned bAuNPs affect their potential as antitumoral agents (Figure 4).

In addition to the main objectives, this work addresses two hypotheses: first, the use of 2,6-dichlorophenolindophenol (DCIP) as an indicator of *P. aeruginosa*’s reducing potential for bAuNPs synthesis, and second, the study of the potential involvement of GSH reductase in bAuNPs synthesis. 

This study aims to contribute to our understanding of bAuNPs synthesized using *P. aeruginosa* and has the potential to advance the development of new and effective treatments for prostate cancer.

In summary, this study does not merely contribute to scientific discourse—it stands as a beacon of progress with the power to redefine how we approach cancer treatment. By harnessing the unique properties of biosynthesized gold nanoparticles, it not only advances our understanding but also sets the stage for a future where innovative therapies in nano-oncology emerge from the synergy of biological processes and cutting-edge research.

## 2. Results and Discussion 

### 2.1. Optimization of the Bacterial Growth Conditions for Maximum Reduction Potential

#### DCIP Method 

**Figure 5:**
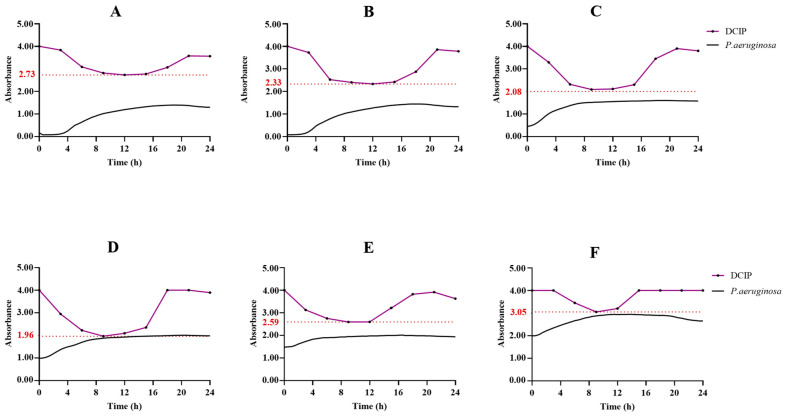
Growth curve of *P. aeruginosa* (black curve) cultured at different optical densities [(**A**) (OD600 = 0.1); (**B**) (OD600 = 0.2); (**C**) (OD600 = 0.5); (**D**) (OD600 = 1.0); (**E**) (OD600 = 1.5); (**F**) (OD600 = 2.0)] and the corresponding supernatant collected at specific times and where DCIP was added (black dots on purple curve). The red dashed line delineates the point at which the absorbance of DCIP undergoes the most pronounced decrease, signifying the juncture wherein the bacterial supernatant manifests its maximal reducing potential.

The results exhibit varying profiles of DCIP reduction across different optical densities (OD600 = 0.1; 0.2; 0.5; 1.0; 1.5; and 2.0). Surprisingly, a pronounced absorbance decay was observed at OD600 = 1.0, with the highest reduction potential consistently occurring during the late exponential growth phase. This reduction potential increased until 9 h (maximum DCIP absorbance decay) and decreased thereafter (Figure 5D). 

**Figure 6:**
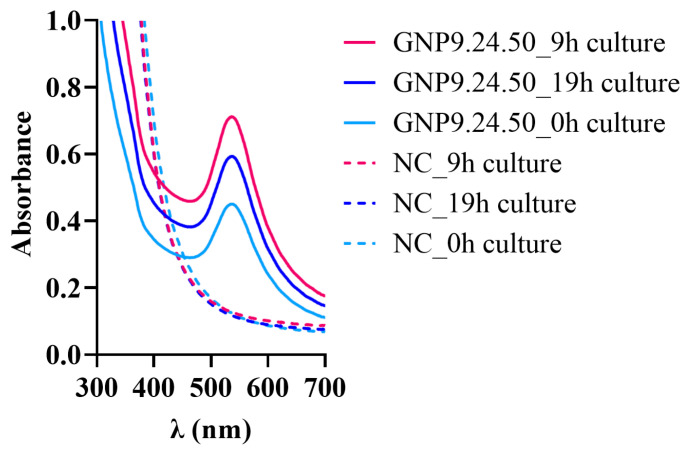
UV-Vis spectra obtained for bAuNPs synthesized at pH 9 for 24 h at 50 °C with PAO1 supernatant collected after 0, 9, and 19 h of culture growth and the respective negative control (NC).

To validate DCIP as an indicator of *P. aeruginosa* reduction potential, bAuNPs were synthesized using culture extracts collected at 0, 9, and 19 h from OD600 = 1.0 cultures. UV-vis spectra analysis revealed that the 9 h culture extract produced the highest bAuNP concentration (0.39 mM), while extracts from 0 and 19 h cultures resulted in lower yields (0.32 mM and 0.37 mM, respectively) (Figure 6).

**Figure 7:**
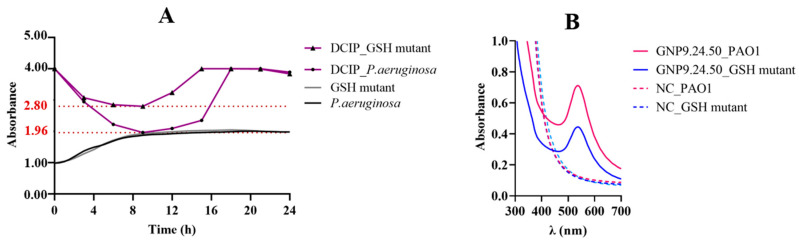
(**A**) *P. aeruginosa* wild type and GSH mutant growth (OD600 = 1.0) and the corresponding supernatant collected at specific times with DCIP addition for *P. aeruginosa* (black dots in purple curve) and for GSH mutant (black triangles in purple curve); (**B**) UV-Vis spectra of bAuNPs synthetized at pH 9.0 for 24 h at 58 °C with *P. aeruginosa* and GSH mutant supernatant and the respective negative control (NC). The red dashed line delineates the point at which the absorbance of DCIP undergoes the most pronounced decrease, signifying the juncture wherein the bacterial supernatant manifests its maximal reducing potential.

To investigate the potential involvement of GSH reductase in bAuNP biosynthesis, a mutated *P. aeruginosa* strain with the GR gene deleted was employed. The deletion of the GR gene resulted in reduced DCIP absorbance decay (Figure 7A) and a lower bAuNP synthesis yield (0.31 mM compared to 0.39 mM) (Figure 7B), supporting the possible role of GSH reductase in the biosynthesis process.

The observed results suggest that metabolites in *P. aeruginosa* can influence DCIP reduction, indicating a potential NADPH-dependent enzyme involvement in bAuNP synthesis. The higher reducing potential at OD600 = 1.0 and 9 h aligns with the late exponential growth phase, which is theoretically characterized by increased production of secondary metabolites [48]. Subsequent bAuNP synthesis experiments with extracts from different growth times supported this hypothesis, with the 9 h extract demonstrating higher yields.

The utilization of a *P. aeruginosa* mutant lacking the GR gene strengthened the role of DCIP as an indicator of reducing potential and suggested the involvement of GSH reductase in bAuNP biosynthesis. However, the incomplete inhibition of bAuNP biosynthesis in the mutant strain implies a potential multifaceted biosynthetic pathway.

Further investigations are warranted to comprehensively understand the intricate mechanisms governing bAuNP biosynthesis and to unravel the interplay between different reducing agents and enzymes in this process [30,48].

### 2.2. Biosynthesis and Characterization of AuNPs

**Figure 8:**
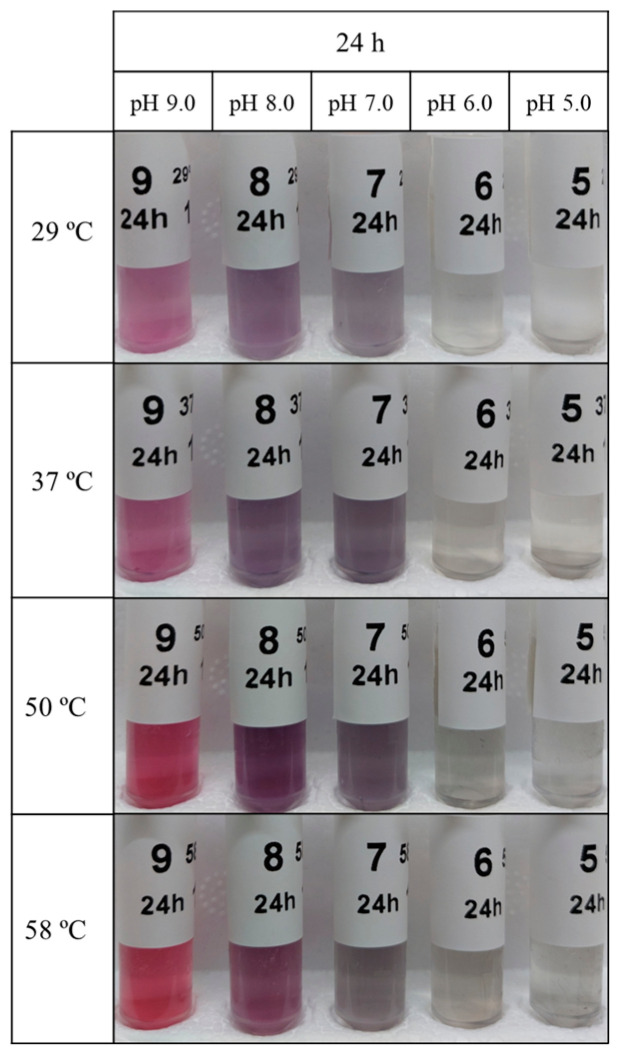
Resulting bAuNPs colloidal solutions synthetized at different pH and temperatures.

By varying a wide range of physicochemical parameters during the reaction process (i.e., pH, time, and temperature), it was possible to observe different patterns in the biosynthesis of AuNPs using *P. aeruginosa*. The synthesis of AuNPs became evident by the color change in the reaction solution, presenting colors ranging from red/pink to purple/blue tones, except for the biosynthesis at pH 5 and 6 that resulted in light-yellow/transparent solutions (Figure 8). 

The synthesis of the bAuNPs was confirmed by UV-Vis, which revealed characteristic peaks in the 500–600 nm wavelength range [49]. UV-Vis spectrometry was used to determine the concentration and aggregation of bAuNPs. The morphological features (shape and size) were obtained using TEM, and the functional groups were determined using ATR-FT-IR. The effects of pH, time, and temperature on the aggregation, concentration, and size of the synthesis of bAuNPs are presented in the following Table 1.

#### 2.2.1. Effect of pH on bAuNP Synthesis

**Figure 9:**
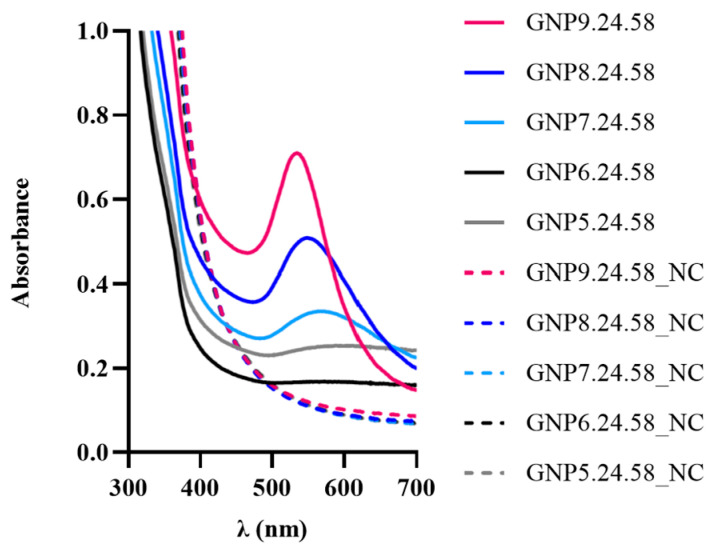
UV-Vis spectra obtained for bAuNPs synthesized at different pH for 24 h at 58 °C and the respective negative control (NC).

**Figure 10:**
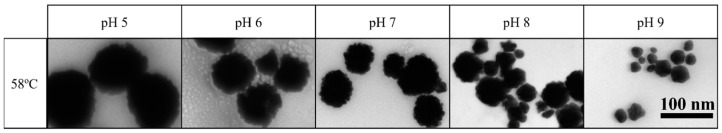
TEM photographs of each bAuNP synthesized at different pH for 24 h at 58 °C (scale bar: 100 nm).

To investigate the pH influence on gold nanoparticle (bAuNPs) biosynthesis across a spectrum from acid to alkaline conditions (pH = 5.0, 6.0, 7.0, 8.0, and 9.0), UV-vis spectroscopy revealed a subtle surface plasmon signal at pH 5 and 6 (GNP5.24.58 and GNP6.24.58), with low bAuNP concentrations (0.17 mM and 0.14 mM) and high aggregation values (0.99) (Table 1). TEM analysis displayed large raspberry-shaped nanostructures at pH 5 and 6 (142.076 ± 34.24 nm and 112.076 ± 26.75 nm) and at pH 7.0 (67.858 ± 33.49 nm). pH increase led to narrower and more intense UV-Vis peaks, with mixed-shape nanoparticles at pH 8.0 (48.791 ± 2.66 nm) and predominantly spherical shapes at pH 9.0 (30,862 ± 13.48 nm) (Figure 9 and Figure 10).

The pH effect on bAuNP synthesis was evident, with a yield increase (0.30 mM and 0.38 mM) at pH 8.0 and 9.0, respectively (Table 1). Notably, pH emerged as a crucial parameter influencing bAuNPs’ size, shape, and yield. The size of nanoparticles decreased with increasing pH, while the reaction yield exhibited an inverse relationship. The significance of pH was underscored by its impact on the reducing power of secondary metabolites and enzymes in the bacterial supernatant. At high pH, OH- ions replaced Cl- ions, hindering potential nuclei growth by repulsions between negatively charged ions and gold ions, maintaining small spherical particles. Lower pH conditions resulted in larger particles, attributed to uncontrolled nucleation from reduced repulsions between AuCl4- and carboxylic groups in the culture extract, aligning with findings by Sneha et al. [50] and Armendáriz et al. [51].

#### 2.2.2. Effect of Time on bAuNP Synthesis

**Figure 11:**
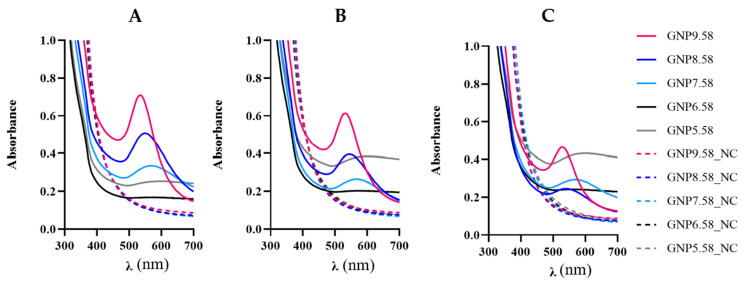
UV-Vis spectra obtained for bAuNPs synthesized at 58 °C for 24 h (**A**), 48 h (**B**), and 72 h (**C**) at different pH and the respective negative control (NC).

The biosynthesis of bAuNPs was investigated over various time intervals (24 h, 48 h, and 72 h) to elucidate the impact on yield, morphology, and aggregation. At pH 9.0, particles initially exhibited small spherical shapes (30.862 ± 13.48 nm) after 24 h (GNP9.24.58). However, at 48 h (GNP9.48.58), an increase in particle size was observed, followed by a decrease at 72 h (GNP9.72.58) while maintaining a spherical shape (29.664 ± 12.21 nm) (Table 1 and Figure 11). The concentration of nanoparticles decreased with time [from GNP9.24.58 (0.32 mM) to GNP9.72.58 (0.30 mM)], accompanied by increased particle aggregation, remaining below 0.4 throughout (Table 1).

Similarly, at pH 8.0, particle growth persisted after 24 h and 48 h [GNP8.24.58 (48.791 ± 22.66 nm) and GNP8.48.58 (58.606 ± 23.88 nm)] (Table 1). Initially showing a raspberry shape, bAuNPs transitioned to a spherical shape after 72 h (GNP8.72.58), with a slight decrease in size (47.340 ± 24.71 nm) (Table 1). Concentration decreased [from GNP8.24.58 (0.30 mM) to GNP8.72.58 (0.17 mM)], while aggregation increased [(from GNP8.24.58 (0.44) to GNP8.72.58 (0.61)]. UV-Vis spectra analysis indicated decreasing peak intensities with time at pH 9.0 and 8.0 (Figure 11).

**Figure 12:**
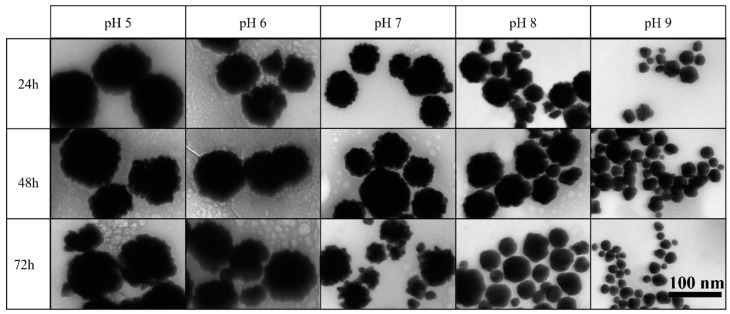
TEM photographs of each bAuNP synthetized at 58 °C at different pH and times (scale bar: 100 nm).

At neutral pH 7.0, a reduction in peak intensity was noted after 48 h (GNP7.48.58) (Figure 11B), persisting at 72 h (GNP7.72.58) (Figure 11C). A slight concentration increase (from 0.16 mM to 0.18 mM) and aggregation rise (from 0.72 to 0.85) occurred from 48 h to 72 h (Table 1). The raspberry shape remained, exhibiting larger spikes approaching a star shape at 72 h (GNP7.72.58) (Figure 12).

At pH 6 and 5, bAuNP synthesis was minimal. However, at pH 5, an increase in concentration was observed over time [from GNP5.24.58 (0.17) to GNP5.72.58 (0.24)] (Table 1). Although the reaction time did not significantly affect bAuNP synthesis, a notable trend emerged. Lower pH levels exhibited increased yield up to 72 h, while higher pH levels demonstrated a decrease over time. This observation may be attributed to the reduced reducing power of secondary metabolites and enzymes at lower pH, resulting in slower nucleation and continuous growth. Conversely, higher pH conditions with increased enzyme and metabolite availability may lead to earlier nucleation, reaching the synthesis limit more rapidly. At 72 h, instability and reduction in concentration and nanoparticle size at pH 8.0 and 9.0 suggest a decomposition phenomenon [52].

#### 2.2.3. Effect of Temperature on bAuNP Synthesis

**Figure 13:**
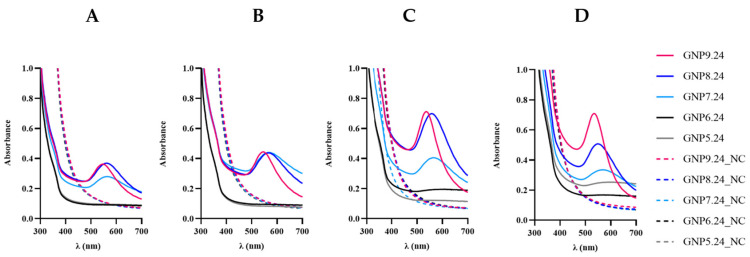
UV-Vis spectrum obtained for bAuNPs synthesized at 29 (**A**), 37 (**B**), 50 (**C**), and 58 °C, (**D**), respectively, for 24 h at different pH and the respective negative control (NC).

To explore the impact of temperature on gold nanoparticle (AuNP) synthesis, experiments were conducted at 29, 37, 50, and 58 °C. Lower temperatures exhibited low-intensity UV-Vis peaks, translating to high aggregation and low concentrations (Table 1.). Surprisingly, at elevated temperatures, particularly 50 °C and 58 °C, pH 9.0 and pH 8.0 demonstrated high-intensity peaks with low aggregation values (Figure 13 and Table 1). However, a slight concentration decrease was observed at 58 °C for pH 9.0 [GNP9.24.58 (0.38 mM)] and a more pronounced decrease at pH 8.0 [GNP8.24.58 (0.30 mM)] compared to 50 °C [GNP9.24.50 (0.39 mM) and GNP8.24.50 (0.36 mM)] (Table 1).

At 37 °C, UV-Vis spectra indicated optimal conditions for AuNP synthesis at neutral pH 7.0 in terms of product yield [GNP7.24.37 (0.26 mM)] (Figure 13A and Table 1). Concentration decreased [GNP7.24.50 (0.24 mM) and GNP7.24.58 (0.22 mM)] with increasing temperature, accompanied by higher aggregation values [GNP7.24.50 (0.80) and GNP7.24.58 (0.72)] (Table 1). Conversely, at pH 5 and 6, higher aggregation occurred at higher temperatures [GNP5.24.58 (1.00) and GNP6.24.58 (1.01)] (Table 1), with UV-Vis spectra showing intensity increases at 50 °C for pH 6.0 (GNP6.24.50) and at 58 °C for pH 5.0 (GNP5.24.58) (Figure 13).

**Figure 14:**
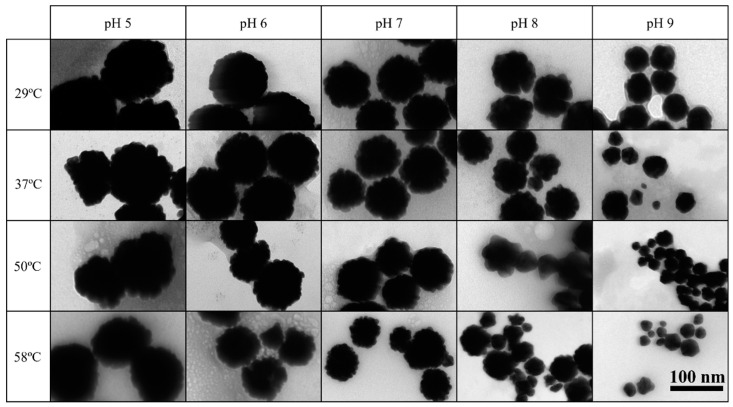
TEM photographs of each bAuNP synthetized for 24 h at different pH and temperatures (scale bar: 100 nm).

TEM analysis generally revealed a slight decrease in particle size with increasing temperature, maintaining raspberry or irregular shapes (Figure 14). An exception was observed at pH 9.0 and pH 8.0 at 58 °C [GNP9.24.58 and GNP8.24.58], presenting cleaner and more rounded surfaces similar to spherical nanostructures (Figure 14). Temperature influenced both size and concentration of synthesized bAuNPs. For pH 7.0, 8.0, and 9.0, 50 °C seemed optimal, yielding the best synthesis results, but instability and particle decomposition increased at 58 °C. At pH 5.0 and 6.0, an increase in bAuNP concentration with rising temperature was observed, suggesting better stability at higher temperatures despite lower concentrations than at higher pH.

### 2.3. Attenuated Total Reflection Fourier-Transform Infrared Spectroscopy—ATR-FT-IR

**Figure 15:**
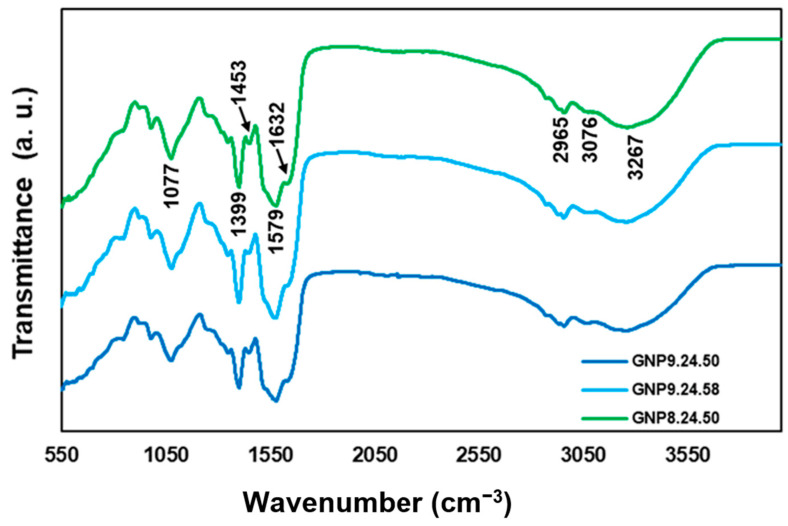
ATR-FT-IR spectra of the gold nanoparticles GNP9.24.50, GNP9.24.58, and GNP8.24.50.

The normalized ATR-FT-IR spectra of the gold nanoparticles GNP9.24.50, GNP9.24.58, and GNP8.24.50 are depicted in Figure 15.

The ATR-FT-IR spectra highlighted in Figure 12 reveal similar spectral properties among the three products: a strong and broad vibration band at 3267 cm^−1^ (attributed to hydroxyl group stretching); a weak vibration at 3076 cm^−1^ (characteristic of N-H stretching); and an absorption peak around 2965 cm^−1^ (associated with C-H stretching in alkanes) [53]. Additionally, ATR-FT-IR spectra show vibrations at 1632 and 1579 cm^−1^ (assigned to amides with carbonyl groups) and absorption bands at 1453, 1399, and 1077 cm^−1^ that can be assigned to the C-O stretching in aromatic and aliphatic amines, respectively [54].

These results affirm the presence of various compounds, indicating the involvement of organic molecules in the bAuNP synthesis process. Notably, the presence of C-O, likely derived from amino acid residues, suggests the coexistence of proteins from the *P. aeruginosa* extract with bAuNPs [55]. This observation raises the hypothesis that these coexisting proteins may influence the internalization rate of bAuNPs in tumor and bacteria cells. Such an interaction could potentially reduce the likelihood of cells recognizing bAuNPs as foreign agents, treating them instead as a form of nutritional supplement.

### 2.4. Cytotoxic Effect Evaluation of bAuNPs in Prostate Cell Lines

The results of the preliminary screening of the bAuNPs’ activity on prostate cell carcinoma are presented in the following subsections of this work. GNP9.24.58 at 10^−3^ mM showed a significant reduction in the viability and a decreasing tendency in the proliferation of PC3 cells, not showing significant activity in HPEpiC cells. As a result, it was the only condition tested in the cellular uptake assay, the injury assay, and the IL-6 ELISA assay.

#### 2.4.1. bAuNP Cellular Uptake Assay

**Figure 16:**
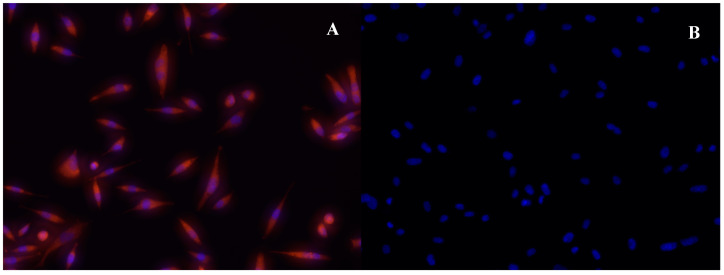
(**A**) Representative microscope photograph of the qualitative PC3 cell uptake assay; in red is rhodamine B-labeled bAuNPs dispersed on the cells and in blue, the cell nucleus marked with DAPI. (**B**) Representative microscope photograph of the control (PC3 cells without addition of rhodamine B-labeled bAuNPs), where only the nuclei of the cells are marked with DAPI, and no fluorescence is captured in red.

The qualitative PC3 cell uptake assay’s representative image displays red fluorescence (Figure 16A), indicating the adsorption of bAuNPs (GNP9.24.58) onto the cells. The blue color corresponds to the cell nucleus, stained with DAPI (Figure 16A). In contrast, the control image solely shows the cell nucleus marked with DAPI, lacking any red fluorescence (Figure 16B). This absence of red fluorescence in the control suggests no adsorption onto the cells in the absence of bAuNPs.

The presence of fluorescent red dots in the rhodamine-B assay allows for the visualization of bAuNPs adhering to PC3 cells, signifying an interaction with the cell membrane. While this observation does not conclusively prove internalization, it does indicate an interaction with the cell membrane. Additionally, the results align with the functional aspect of bAuNPs, as the coating with rhodamine-B requires amine groups on the bAuNPs’ surface. The fluorescence observed supports the ATR-FT-IR results, reinforcing the potential coexistence of amine groups with the bAuNPs [56].

#### 2.4.2. Viability, Proliferation, and Injury Assay

**Figure 17:**
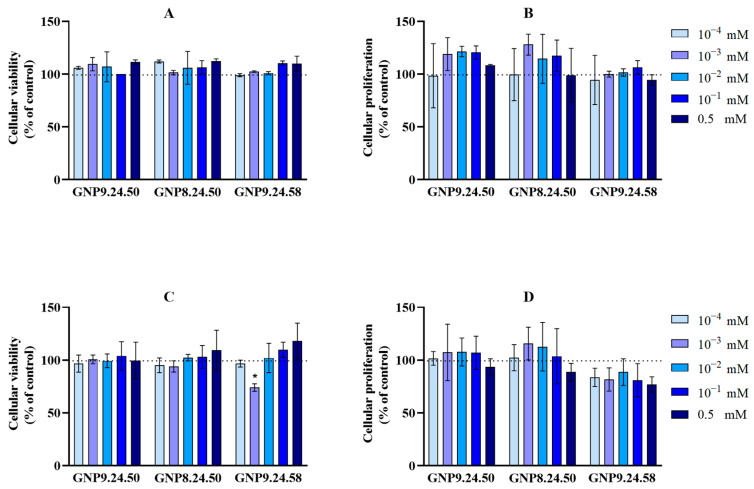
Cellular viability (**A**) and proliferation (**B**) as percentage of control for **PC3 cells** after 24 h treatment with GNP9.24.50, GNP8.24.50, and GNP9.24.58 at different concentrations. Cellular viability (**C**) and proliferation (**D**) as percentage of control for **HPEpiC cells** after 24 h treatment with GNP9.24.50, GNP8.24.50, and GNP9.24.58 at different concentrations (* *p* < 0.05). Legend: 0.5 to 10^−4^ mM: 0.5 mM to 0.0001 mM AuNPs.

The viability and proliferation assays were conducted after a 24 h treatment with bAuNPs (GNP9.24.50, GNP8.24.50, and GNP9.24.58). In all analyzed conditions, no dose-dependent relationship was observed. Regarding the impact of AuNPs on HPEpiC cells, no significant differences were found (Figure 17). However, an increasing tendency in cell proliferation was noticed after treatment with GNP9.24.50 and GNP8.24.50 in the concentration range of 0.5–10^−3^ mM (Figure 17B). For PC3 cells, only GNP9.24.58 at 10^−3^ mM proved to have a statistically significant (*p* < 0.05) effect on cell viability (74%) (Figure 17C), also showing a decreasing tendency in cell proliferation at the same concentration (Figure 17D).

**Figure 18:**
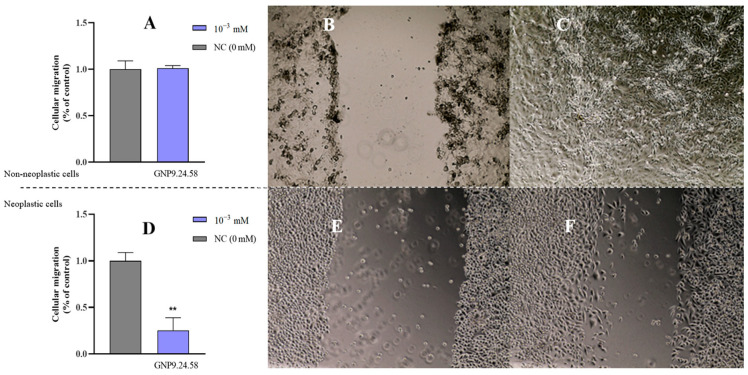
Cellular migration of **HPEpiC cells** as percentage of control (**A**) after 24 h of treatment with GNP9.24.58 (10^−3^ mM) (blue bar) and after 24 h of growth without bAuNPs (NC, gray bars) (** *p* < 0.01). Microscope photographs denoting the difference between 0 (**B**) and 24 h (**C**) of treatment with GNP9.24.58 (10^−3^ mM) of the **HPEpiC cells**. Cellular migration of **PC3 cells** in relation to the control (**D**) after 24 h of treatment with GNP9.24.58 at (10^−3^ mM) and after 24 h of growth without bAuNPs (NC, gray bars). Microscope photographs denoting the difference between 0 (**E**) and 24 h (**F**) of treatment with GNP9.24.58 at (10^−3^ mM) of **PC3 cells**. Legend: 10^−3^ mM: 0.001 mM AuNPs; NC (0 mM): negative control, without AuNPs.

The results of the injury assay are depicted in Figure 18. GNP9.24.58 (10^−3^ mM) did not produce any effect on the migration of HPEpiC cells after 24 h of treatment (Figure 18A), showing complete wound closure (Figure 18C). Contrarily, when the same bAuNP was applied in PC3 cells, at the same concentration, prevention of wound closure was observed (Figure 18F). In PC3 cells, cellular migration was significantly lower (*p* < 0.01) in relation to the control (75%) (Figure 18D).

Among the three types of nanoparticles tested, GNP9.24.58 was the one that produced a higher anti-viability and antiproliferation effect on PC3 cells without affecting HPEpiC cells. These results align with observations from other studies, which attribute greater potential to more spherical and smaller nanoparticles [36,37]. At 10^−3^ mM, a statistically significant viability decrease (*p* < 0.05) was observed in PC3 cells. Although the same statistical significance was not noted for cell proliferation, there was a downward trend compared to the control line at this concentration, indicating that, possibly, although viability is significantly reduced, there may not be total cell death, allowing surviving cells to continue proliferating. However, after injury, there was a reduction in the migratory capacity by about 75% compared to the control. This fact, from a clinical point of view, becomes more preponderant. Nair et al. found that patient survival is significantly more strongly associated with predicted tumor migration levels than with predicted tumor proliferation levels [53]. 

The results presented in Figure 16 suggest that an increased concentration of bAuNPs does not necessarily lead to a heightened antitumoral effect. This highlights the importance of selecting an optimal concentration. Further research is crucial to fully understand the mechanisms underlying the antitumorigenic activity of AuNPs and to optimize their application as antitumoral agents.

#### 2.4.3. Human Interleukin 6 (IL-6) ELISA Assay

**Figure 19:**
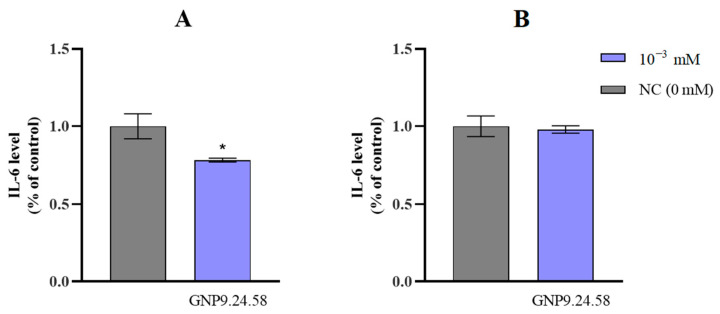
IL-6 levels of HPEpiC cells (**A**) and PC3 cells (**B**) as percentage of control after 24 h of treatment with GNP9.24.58 at (10^−3^ mM) (blue bars) and after 24 h of growth without bAuNPs (NC, gray bars) (* *p* < 0.05). Legend: 10^−3^ mM: 0.001 mM AuNPs; NC (0 mM): negative control, without AuNPs.

As a complementary analysis, IL-6 levels in PC3 and HPEpiC cultures were quantified. GNP9.24.58 (10^−3^ mM) had no discernible effect on IL-6 levels in HPEpiC cells after 24 h of treatment (Figure 19A). In contrast, a statistically significant (*p* < 0.05) reduction (20%) was observed for PC3 cells (Figure 19B).

While IL-6 is a crucial protein in the regulation of the inflammatory process, a decrease in its levels after treatment with AuNPs does not necessarily directly translate to a reduction in tumor aggressiveness. It is essential to consider that IL-6 is part of a complex regulatory mechanism of the inflammatory response involving other key players. Nevertheless, this protein has been associated with tumor growth at specific stages of tumor progression. Examining the results obtained (Figure 18B), a decreasing tendency in IL-6 levels is evident, as observed by Al-Trad B. et al. in their study on the effect of AuNPs against induced benign prostate carcinoma in rats [4], while in HPEpiC cells, the levels remain stable. Further studies are required to fully understand the mechanisms behind the observed potential of bAuNPs to downregulate inflammation in PC3 cells.

## 3. Materials and Methods

### 3.1. AuNP Biosynthesis—Optimization and Characterization 

#### 3.1.1. Chemicals

In the study, the specific chemicals and reagents used were as follows: gold(III) chloride, and HAuCl_4_ (ACS reagent), which was obtained from Sigma Aldrich in St. Louis, MO, USA, and used without further purification. DCIP was sourced from Sigma-Aldrich, Merck KGaA, located in Missouri, USA. The 1% penicillin/streptomycin solution used was from Gibco, a product of Life Technologies with catalog number 10,270, in the USA. For fluorescent staining and imaging purposes, 4′,6-diamidino-2-phenylindole DAPI at a concentration of 10 ng/mL was obtained from Thermo Fisher Scientific in the USA. It should be noted that all other reagents utilized in this study were of analytical grade.

#### 3.1.2. Pseudomonas Aeruginosa Strain and Maintenance 

*Pseudomonas aeruginosa* (PAO1, ATCC 15692) was provided and maintained at the Laboratory of Medical and Industrial biotechnology (LaBMI), Porto Research, Technology & Innovation Center (PORTIC), Porto, Portugal. Bacteria were routinely cultured in trypticase soy agar (TSA) plates, where the fluorescent pigment pyoverdine typically produced by *P. aeruginosa* strains was observed.

#### 3.1.3. Optimization of the Bacterial Growth Conditions for Maximum Reduction Potential

**Figure 20:**
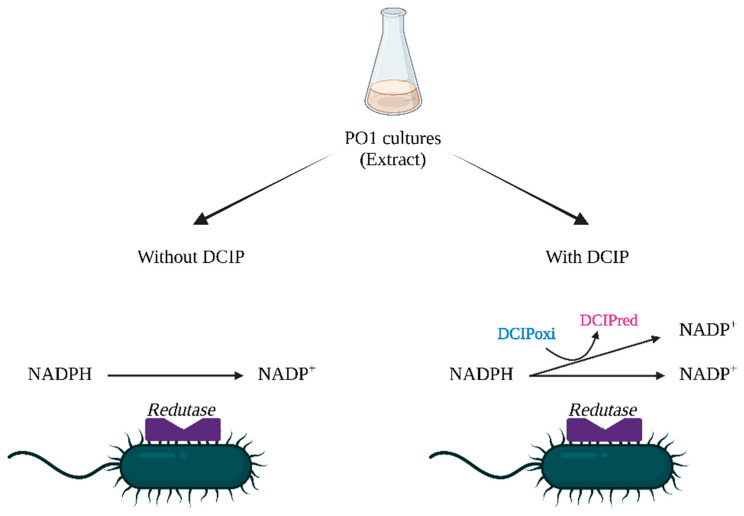
Putative mechanism for the proposed DCIP method. On the left is NAPH-dependent reductase present in *P. aeruginosa* extract without the addition of DCIP. On the right is an alternative route for NADPH molecule in *P. aeruginosa* extract being oxidized and reducing DCIP from blue to red.

DCIP (Sigma-Aldrich, Merck KGaA, St. Louis, MO, USA) is a compound that can be directly reduced by NAPH, the final reaction products of two electron reduced forms of DCIP and NADP+ (Equation (1)) [57], with a change in color from blue to red, easily denoted by an absorbance decay at 600 nm (OD_600_). Bearing in mind that NADPH may be involved in the synthesis of nanoparticles by interacting with reductases, DCIP is suggested as an indicator of *P. aeruginosa* reduction potential (Figure 20). Experiments were conducted to determine at which optical densities (OD) of the bacterium the absorbance decay of DCIP is most pronounced over time, and to compare the efficiency of the bacteria extract in the synthesis of bAuNPs.

**DCIP**_oxi_ + **NADPH** → **DCIP**_red_ + **NADP**^+^ (1)

Isolated colonies of *P. aeruginosa* were cultured in tryptic soy broth (TSB) at the following optical densities (OD_600_): 0.1, 0.2, 0.5, 1, 1.5, and 2.0 at 37 °C. Each culture was centrifuged for 10 min at 4000 rpm. The supernatant was recovered, and DCIP (20 mg/mL) was added, with the absorbance decay being measured by spectrophotometry at 600 nm (OD_600_) after 24 h. The process was repeated with a time interval of 3 h during 24 h of culture growth. A kinetic measurement of *P. aeruginosa* culture in the same OD was run in parallel for a 24 h period at 37 °C with a time interval of 30 min and with no DCIP addition. All measurements were carried out in triplicate. 

#### 3.1.4. DCIP Method as an Indicator of PAO1 Reduction Potential and Investigation of GR Gene’s Possible Involvement in AuNP Biosynthesis 

To confirm the hypothesis of DCIP as an indicator of PAO1 reduction potential in bAuNP biosynthesis, the optical density was adjusted to 1.0 (OD_600_) and the culture was grown in 100 mL of TSB at 37 °C. Cultures were maintained at 150 rpm. The supernatant was recovered by centrifugation at 4000 rpm for 10 min at four different culture times: 0, 9, and 19 h. After, a solution of chloroauric acid (HAuCl_4_, 50 mM) (Sigma-Aldrich, Merck KGaA, St. Louis, Missouri, USA) was mixed with the cell free supernatant. The pH was kept at 9.0 by using a NaOH solution (0.1 M). All solutions were mixed in triplicate and maintained at 50 °C for 24 h. Following that, a *P. aeruginosa* strain with a deletion in the GR gene (mutant) was employed to investigate the possible involvement of GSH reductase in the biosynthesis of AuNPs. This mutant was also grown in 100 mL of TSB for 37 °C and kept under stirring at 150 rpm. The supernatant was recovered at 9 h. Again, a solution of HAuCl_4_ (50 mM) was added to the cell free supernatant and the pH was adjusted to 9.0 with NaOH (0.1 M) solution. The reaction was performed in triplicate at 50 °C for 24 h. Together with the experimental tubes, solutions also subjected to the same pH adjustment but without the addition of chloroauric acid were used as control. 

#### 3.1.5. Biosynthesis of AuNPs in Different Physicochemical Conditions

To evaluate the effect of different physicochemical parameters, the biosynthesis of bAuNPs was carried out under different reaction conditions. *P. aeruginosa* was again cultured at OD_600_ = 0.1 in 100 mL of TSB and maintained at 150 rpm for 9 h. The supernatant was recovered by centrifugation at 4000 rpm. After, a HAuCl4 solution (50 mM) was mixed with the cell free supernatant.

Effect of pH on bAuNP synthesis: the pH of the cell free extract was varied at 5.0, 6.0, 7.0, 8.0, and 9.0 with different reaction temperatures (29, 37, 50, and 58 °C) and different reaction times (24, 48, and 72 h) by adding 0.1M HCl or 0.1M NaOH until it reach the required pH.

Effect of reaction temperature and time on bAuNP synthesis: the reaction mixtures obtained by varying the pH were maintained in the experimental tubes and incubated in a rotary shaker at 150 rpm for varied time intervals of 24, 48, and 72 h at different reaction temperatures (29, 37, 50, and 58 °C).

Control solutions (without the addition of HAuCl4) were also mixed in the experimental tubes and subjected to the same reaction conditions. Sample identity (ID) indicates the physicochemical parameters for the synthesis of bAuNPs. For example, in sample ID GNP9.24.58, GNP indicates gold nanoparticles, 9 indicates pH 9.0, 24 indicates reaction time in hours, and 58 indicates incubation temperature.

#### 3.1.6. Characterization of Biosynthesized bAuNPs

##### UV–Visible Spectroscopy (UV-Vis)

To confirm the presence of AuNPs, the excitation spectra of the samples were measured by UV-Vis using the Multiskan SkyHigh spectrophotometer (Thermo Fisher ScientTific, Waltham, MA, USA). The pellets of control and AuNP solutions were resuspended in DEPC-treated water (Thermo Fisher Scientific, Waltham, MA, USA) and mixed by vortex, and the excitation spectra were recorded in a wavelength range from 300 to 700 nm. All measurements were conducted in a quartz cuvette (1 mm). Concentrations of gold ions that were reduced to AuNPs in the biosynthesis process were calculated following Scarabelli et al.’s [58] correlation, where, from the absorbance at 400 nm and for a 1 mm cuvette, an absorbance of 1.2 (OD_400_) corresponds to [Au^0^] = 0.5 mM:

(2) $$\frac{1.2}{A\;400\;\mathrm{nm}}=\frac{0.5\;\mathrm{mM}}{x}$$

**Also, UV**-Vis data were used to obtain the aggregation values, as described by Ye et al. [9]. The ratio between the absorbance at 650 nm (aggregated AuNPs) and the absorbance at 530 nm (dispersed AuNPs) was used to express the molar ratio of aggregated to dispersed AuNPs:

(3) $$Agregation=\frac{A\;650\;\mathrm{nm}}{A\;530\;\mathrm{nm}}$$

##### Transmission Electron Microscopy (TEM)

A total of 10 µL of each sample was mounted on carbon film-coated mesh nickel grids and left to stand for 2 min. The excess liquid was removed with filter paper from all samples, and the grids were observed in a JEM 1400 TEM (JOEL Ltd., Tokyo, Japan) with an accelerating voltage of 80 kV. Images were digitally recorded using a CCD digital camera (Orious 1100W Tokyo, Japan). After, images were analyzed using ImageJ V1.53 (U. S. National Institutes of Health, Bethesda, MD, USA) software to assess the mean particle size.

##### Attenuated Total Reflection Fourier-Transform Infrared Spectroscopy (ATR-FT-IR)

The gold nanoparticles presenting higher yields (GNP9.24.50, GNP8.24.50, and GNP9.24.58) were subjected to ATR-FT-IR analysis. The ATR-FT-IR analyses were performed using the Frontier™ MIR/FIR spectrometer (PerkinElmer, Waltham, MA, USA) in a scanning range of 550–4000 cm^−1^ for 16 scans at a spectral resolution of 4 cm^−1^. 

### 3.2. bAuNPs against Prostate Cancer—Cytotoxic Effect Evaluation of the bAuNPs in Prostate Cell Lines

In the following subsections of this work, we present the methods for preliminary screening of the bAuNPs’ activity on prostate cell carcinoma. To study the effects of the bAuNPs, two different cell models were used: the neoplastic prostate cell line PC3, which is derived from a cancerous prostate tissue, and the normal epithelial cell line HPEpiC, which is derived from healthy prostate tissue. In order to evaluate the activity of the bAuNPs, a series of in vitro assays were performed, including assessments of cell viability, proliferation, migration, and IL-6 levels. GNP9.24.50, GNP8.24.50, and GNP9.24.58 were tested. These bAuNP conditions were chosen due to their consistently high synthesis yields during the synthesis process.

#### 3.2.1. Cell Culture and Treatments

PC3 and HPEpiC were seeded in RPMI 1640 medium (VWR, Biowest, P0860-N10L, Brandeton, FLA, USA) supplemented with 10% fetal bovine serum (FBS, Gibco, Life technologies, 10270, Waltham, MA, USA) and 1% penicillin/streptomycin (Gibco, Life technologies, 10270, Waltham, MA, USA). The cells were maintained at 37 °C in a humidified chamber containing 5% CO_2_. Treatments were performed using three different bAuNPs, GNP9.24.50, GNP8.24.50, and GNP9.24.58, at a final concentration range of 0.5–10^−4^ mM, and the dilutions were made using the serial dilution method, with the serum free cell culture medium being the solvent used. GNP9.24.58 at 10^−3^ mM final concentration was the only condition used in the cellular uptake, injury, and IL-6 ELISA assays.

#### 3.2.2. bAuNP Cellular Uptake Assay

Fluorescence microscopy was used for qualitative examination of the uptake of rhodamine B fluorescent-labeled bAuNPs by PC3 cell lines. In the bAuNP suspension, the rhodamine B (RhoD) solution was added followed by stirring for 24 h at 25 °C in a dark room. Then, RhoD-loaded bAuNPs were recovered by centrifugation at 5000 rpm for 10 min. The cells were grown in cover slips and treated with rhodamine B-loaded bAuNPs, and non-treated cells were used as control. After 24 h, the cells were washed with phosphate-buffered saline (PBS). Finally, nuclei were marked with 4′,6-diamidino-2-phenylindole (DAPI). Before analysis, cells were fixed using paraformaldehyde (3.7%), followed by three washing steps. After the washing steps, 300 µL of DAPI (10 ng/mL, Thermo Fisher Scientific, Waltham, MA, USA) was added and the culture was incubated for 10 min at 25 °C. Rhodamine B-labeled bAuNP uptakes were observed using a fluorescence microscope (EVOS M7000 microscope, Thermo Fisher Scientific, Waltham, MA, USA). 

#### 3.2.3. Viability Assay

The 3-[4,5-dimethylthiazol-2-yl]-2,5 diphenyl tetrazolium bromide (MTT) assay (Life Technologies, Thermo Fisher Scientific, Waltham, MA, USA) was carried out following the manufacturer’s instruction. In brief, 1.0 × 10^5^ cells/mL (PC3) or 2.5 × 10^5^ cells/mL (HPEpiC) were seeded in a 96-well plate and grown until 80% confluence. Each bAuNP was incubated for 24 h in cell culture. After the washing step with warm PBS, 0.5 mg/mL of MTT was incubated for 2 h at 37 °C. The absorbance was then measured with a microplate reader at 570 nm (Multiskan SkyHigh, Thermo Fisher Scientific, Waltham, MA, USA). 

#### 3.2.4. Proliferation Assay

The proliferative potential of the cells was evaluated using the Roche Diagnostics BrdU kit and was carried out for the colorimetric bromodeoxyuridine (BrdU) assay (Rotkreut, Switzerland). Briefly, PC3 (1.0 × 10^5^ cells/mL) and HPEpiC (2.5 × 10^5^ cells/mL) were seeded into a 96-well flat-bottomed tissue culture plate until reaching 80% confluence. The cell culture was seeded with 0.01 mM BrdU for 24 h during the treatment period with bAuNPs. After binding to the BrdU integrated into the newly synthesized DNA using an antibody–bromodeoxyuridine–peroxidase (anti-BrdU-POD) solution, the supernatant was removed, and the wells were rinsed three times with a 1× washing solution. The substrate solution was incubated in the dark at room temperature for 10 min to detect immune complexes. The reaction product was quantified by measuring the absorbance at 660 nm using a microplate reader (Multiskan SkyHigh, Thermo Fisher Scientific, Waltham, MA, USA). 

#### 3.2.5. Injury Assay

PC3 (4.0 × 10^5^ cells/mL) and HPEpiC (6.0 × 10^5^ cells/well) cells were seeded into a 6-well flat-bottomed tissue culture plate and grown to confluence before being injured with a pipette tip (200 µL). Following that, the bAuNPs were added to the cultures, and the wounded cell monolayer was photographed with an inverted microscope at a magnification of 10× (Nikon Instruments Inc., Melville, NY, USA). The migration distance was scanned 24 h after treatment, and the scratch closure was determined using Image J V1.53 (U. S. National Institutes of Health, Bethesda, MD, USA) software by measuring the damage width.

#### 3.2.6. Human Interleukin 6 (IL-6) ELISA Assay

The detection and quantification of IL-6 were conducted using a solid-phase sandwich enzyme-linked immunosorbent assay (ELISA). Firstly, PC3 (4.0 × 105 cells/mL) and HPEpiC (6.0 × 105 cells/well) cells were seeded into a 6-well flat-bottomed tissue culture plate and grown to confluence, followed by the addition of GNP9.24.58 (10^−3^ mM final concentration), with the culture medium being collected after 24 h. The supernatant was used to perform the ELISA assay (Invitrogen, Thermo Fisher Scientific, Waltham, MA, USA), following the manufactures instructions. In brief, 100 µL of the sample (culture medium) and 50 µL of the Hu IL-6 biotin conjugate solution were added to each well of a 98-well plate. After 2 h of incubation, the solution was removed, and each well was washed 4 times with 1× washing solution. After 2 h of incubation, the solution was removed and 100 µL of 1× Streptavidin-HRP solution was added and incubated for 30 min; the solution was then removed, and wells were washed 4 times. Subsequently, stabilized chromogen was added to each well and incubated for 30 min at room temperature in the dark. Finally, 100 µL of Stop Solution was added and absorbance was measured at 450 nm. 

## 4. Conclusions

In conclusion, our exploration into *P. aeruginosa*-mediated biosynthesis of gold nanoparticles, coupled with the innovative application of DCIP screening, has unveiled a pathway to optimize culture conditions for enhanced yields. Notably, the pivotal role of pH, temperature, and time in influencing synthesis outcomes has been delineated. The synthesis of bAuNPs at pH 9.0 for 24 h at 58 °C showcased superior yields and yielded nanoparticles with desirable characteristics.

Moreover, our findings, marked by distinctive absorption bands in ATR-FT-IR spectra, suggest a potential avenue for enhancing bAuNPs’ biocompatibility and cellular internalization. The demonstrated impact on tumor cell viability, proliferation, migration, and IL-6 levels positions GNP9.24.58 as a promising candidate for targeted and effective prostate cancer therapy. The simplicity, high yields, and biocompatibility achieved through *P. aeruginosa*-driven synthesis open avenues for green therapeutic strategies. This study not only expands the scientific understanding of nanoparticle synthesis but also charts a course toward innovative and sustainable approaches in nano-oncology.

## Figures and Tables

**Table 1:** Effect of different physicochemical parameters on the biosynthesis of bAuNPs.

	pH	Time (h)	Temp. (°C)	Agreggation (650/530 nm)	Concent. (mM)	TEM Size (nm)
Effect of pH						
GNP9.24.58	9.0	24	58	0.23	0.38	30.862 ± 13.48
GNP8.24.58	8.0	24	58	0.44	0.30	48.791 ± 22.66
GNP7.24.58	7.0	24	58	0.72	0.22	67.858 ± 33.49
GNP6.24.58	6.0	24	58	0.99	0.14	112.076 ± 26.75
GNP5.24.58	5.0	24	58	0.99	0.17	142.076 ± 34.24
Effect of pH and time						
GNP9.24.58	9.0	24	58	0.23	0.38	30.862 ± 13.48
GNP9.48.58	9.0	48	58	0.29	0.34	35.264 ± 13.48
GNP9.72.58	9.0	72	58	0.32	0.32	29.664 ± 12.21
GNP8.24.58	8.0	24	58	0.44	0.30	48.791 ± 22.66
GNP8.48.58	8.0	48	58	0.52	0.24	58.606 ± 23.88
GNP8.72.58	8.0	72	58	0.61	0.17	47.340 ± 24.71
GNP7.24.58	7.0	24	58	0.72	0.19	63.233 ± 33.49
GNP7.48.58	7.0	48	58	0.80	0.16	63.532 ± 34.63
GNP7.72.58	7.0	72	58	0.85	0.18	63.942 ± 30.59
GNP6.24.58	6.0	24	58	0.99	0.14	112.076 ± 26.75
GNP6.48.58	6.0	48	58	0.99	0.21	112.428 ± 33.32
GNP6.72.58	6.0	72	58	0.97	0.22	112.501 ± 32.39
GNP5.24.58	5.0	24	58	0.99	0.17	142.076 ± 34.24
GNP5.48.58	5.0	48	58	1,00	0.22	142.82 ± 28.86
GNP5.72.58	5.0	72	58	1,00	0.24	142.88 ± 29.49
Effect of pH and temperature						
GNP9.24.29	9.0	24	29	0.50	0.18	56.342 ± 12.92
GNP9.24.37	9.0	24	37	0.47	0.24	47.811 ± 16.01
GNP9.24.50	9.0	24	50	0.33	0.39	42.063 ± 16.86
GNP9.24.58	9.0	24	58	0.23	0.38	30.862 ± 13.48
GNP8.24.29	8.0	24	29	0.84	0.20	75.854 ± 11.85
GNP8.24.37	8.0	24	37	0.88	0.24	73.300 ± 16.01
GNP8.24.50	8.0	24	50	0.49	0.36	54.894 ± 35.50
GNP8.24.58	8.0	24	58	0.44	0.30	48.791 ± 22.66
GNP7.24.29	7.0	24	29	0.82	0.17	88.072 ± 20.91
GNP7.24.37	7.0	24	37	0.81	0.26	87.296 ± 22.78
GNP7.24.50	7.0	24	50	0.80	0.24	76.481 ± 32.92
GNP7.24.58	7.0	24	58	0.72	0.22	67.858 ± 33.49
GNP6.24.29	6.0	24	29	0.96	0.05	124.816 ± 28.53
GNP6.24.37	6.0	24	37	0.97	0.06	122.386 ± 56.61
GNP6.24.50	6.0	24	50	1.00	0.15	120.141 ± 44.45
GNP6.24.58	6.0	24	58	1.01	0.14	112.077 ± 26.75
GNP5.24.29	5.0	24	29	0.95	0.08	154.172 ± 37.56
GNP5.24.37	5.0	24	37	0.94	0.08	145.926 ± 31.49
GNP5.24.50	5.0	24	50	0.97	0.12	144.814 ± 36.36
GNP5.24.58	5.0	24	58	1.00	0.17	142.076 ± 34.24

## Data Availability

All data that support the findings of this study are available within the article or from the corresponding authors upon reasonable request.
